# Performance analysis of a dedicated breast MR-HIFU system during ablation of breast tumors in patients

**DOI:** 10.1186/2050-5736-3-S1-P43

**Published:** 2015-06-30

**Authors:** Roel Deckers, Baudouin Denis de Senneville, Laura Merckel, Gerald Schubert, Floor Knuttel, Max Kohler, Willem Mali, Chrit Moonen, Maurice van den Bosch, Lambertus W Bartels

**Affiliations:** 1 University Medical Center Utrecht, Utrecht, Netherlands; 2 Philips Healthcare, Vantaa, Finland

## Background/introduction

We have recently conducted a clinical phase I study to assess the safety and spatial accuracy and precision of a newly developed dedicated MR-HIFU system for breast tumor ablation. In this system the breast is placed in a water filled cup, which is surrounded by an arc of ultrasound transducers.[1] This lateral sonication approach significantly reduces the risks of heating tissues in the near field (i.e. skin) and in the far field (i.e. ribs, heart and lungs), which in turn allows the usage of higher powers and/or longer sonication durations in a larger treatment area. Here, we report on the performance of real-time MR thermometry and on the accuracy and precision of the dedicated system.

## Methods

All treatments were performed on a dedicated breast MR-HIFU system (Philips Healthcare, Vantaa, Finland) integrated with a clinical 1.5-T MRI scanner (Achieva, Philips Healthcare, Best, The Netherlands). Ten female patients with pathologically proven invasive breast cancer after large-core needle biopsy were included. The patients were under procedural sedation during the complete HIFU procedure. The tumor tissue was deliberately partially ablated, to be able to use the remaining tumor tissue for histopathological characterization and staging and to allow for histological analysis of viable *versus* ablated tumor tissue. At least 48 hours after MR-HIFU treatment the tumor was removed surgically. The total number of sonications per patient (1-5, excluding test sonications) varied. Fat-suppressed segmented Echo Planar Imaging was performed for PRFS-based thermometry. A Look-Up-Table (LUT)-based correction method was used to correct on-line for respiration-induced magnetic field fluctuations.[2] The performance of the correction method was assessed in absence of HIFU heating. The temporal standard deviation (SD) in temperature maps calculated before and after applying the correction method and the number of dynamics needed before switching to the intervention phase were used as a measure of performance. The targeting accuracy and precision of the system was assessed using MR thermometry data. For each sonication the trajectory of the center of mass of the heating pattern in the focal area over time was calculated. The standard deviation of the distribution of the distances between the center of mass in time and the mean center of mass was defined as precision. The distance between the mean center of mass and the planned treatment position was defined as accuracy.

## Results and conclusions

The LUT-based correction method significantly improved the precision of MR thermometry, the mean temperature SD decreased from 8.0 °C to 2.9 °C for the coronal slice and from 8.3 °C to 2.9 °C for the sagittal slice. On average, 67 images were required to complete the learning phase for the LUT-based correction method, which corresponds to 150 seconds. The mean accuracy was equal to 2.4 mm in the coronal as well as the sagittal image. The mean precision was equal to 1.4 mm and 1.9 mm in the coronal and sagittal image, respectively. Furthermore, the necrotic areas observed on histology corresponded to the number and location of the sonications performed. In conclusion, the precision of the MRT after respiration-induced temperature error correction is high enough for image guidance during tumor ablation. The dedicated breast MR-HIFU system allows for the ablation of breast cancer with a high spatial accuracy and precision.
